# Care of the Hair

**Published:** 1896-06

**Authors:** 


### Care of the Hair.

"If you want to keep your hair in good condition you should remember to brush, brush, brush and brush again," says one who has made the care of the hair a life study. "Brushing is absolutely the only means one can use to make the hair glossy, clean and perfectly healthy. Women often ask me what to use to increase the growth of their hair. The hair is very obedient to the treatment it receives, and if that is good and regular, and one is in good health, the hair needs no tonic."

"To what do you attribute baldness?" she was asked.

"It is almost an unfailing sign of intellectual activity. Brain workers are most liable to it. People of the laboring classes who gain their bread with their hands are generally exempt from baldness until they have passed beyond the sixtyyear mark. Just why this is so, why the workingman who takes no particular care of his head-thatch would be able to preserve it longer than the man who spends much time in having it brushed and shampooed, is a mystery not yet explained. The mane of a thoroughbred horse even is thinner than that on the neck of his brother who drags a dray or horse-car."—*N. Y. Tribune.*

